# Conscious Sedation Versus General Anesthesia in Transcatheter Aortic Valve Replacement: A Cost and Outcome Analysis

**DOI:** 10.7759/cureus.4812

**Published:** 2019-06-03

**Authors:** Mansoor Ahmad, Jay N Patel, Sharath C Vipparthy, Chirag Divecha, Pablo X Barzallo, Minchul Kim, Steven C Schrader, Marco Barzallo, Sudhir Mungee

**Affiliations:** 1 Internal Medicine, University of Illinois College of Medicine at Peoria, Peoria, USA; 2 Cardiology, University of Illinois College of Medicine at Peoria, Peoria, USA; 3 Anesthesiology, University of Illinois College of Medicine at Peoria, Peoria, USA

**Keywords:** transcatheter aortic valve replacement (tavr), sedation, anesthesia

## Abstract

Background

Transcatheter aortic valve replacement (TAVR) has emerged as an alternative treatment for aortic stenosis in patients who are at moderate to high risk for surgical aortic valve replacement. The use of conscious sedation (CS) as compared with general anesthesia (GA) has shown better clinical outcomes for TAVR patients. Whether CS has any cost-benefit is still unknown. We analyze our local TAVR registry with a focus on the cost comparison between CS and GA for the TAVR population.

Methods

It is a retrospective chart review of 434 patients who received TAVR at our local center from December 2012 to April 2018. Patients who had their procedure aborted and those requiring a cardiopulmonary bypass or surgical conversion (16 patients) were excluded. The final sample size was 418. Patients were divided into two groups based on whether they received CS or GA. Primary outcomes were intensive care unit (ICU) hours, length of stay in hospital, readmission, or death at 30 days. The secondary outcome was the cost of TAVR admission. The cost was divided into direct and indirect costs. The student's T-test and chi-square tests were used for continuous and categorical variables, respectively. Adjusted logistic regression and multivariate analyses were run for primary and secondary outcomes.

Results

Of the 418 patients (age: 80.9±8.5, male: 52%) CS was given to 194 patients (46.4%) while GA was given in 224 patients(53.6%). The GA group had comparatively older age (81.8 vs. 80.0; p=0.03) and a higher average Society of Thoracic Surgery (STS) score (8.4 vs 5.7; p<0.001). Patients who received CS had a significantly shorter ICU stay (31.5 vs. 41.6 hours, p<0.001) and total days in the hospital (2.9 vs. 3.8 days, p=0.01). Readmission and mortality at 30 days were not different between the groups. There was no statistical difference in cost between the two groups ($72,809 vs. $71,497: p=0.656).

Conclusion

Using CS compared with GA improves morbidity for TAVR patients, in the form of ICU stay and the total length of stay in hospital. We did not find a significant difference in the cost of TAVR admission between CS and GA.

## Introduction

Transcatheter aortic valve replacement (TAVR) is a relatively new procedure that was initially introduced for high-risk patients in 2011 [1]. Since that time, the adoption of TAVR has expanded to commercial use for intermediate and low-risk patients. Advancements in device design and delivery systems, as well as increased procedural experience, have led to similar rates of death and stroke in TAVR when compared with surgical aortic valve replacement (SAVR) [2-4]. As a result, the utilization of TAVR is only slated to increase exponentially once this becomes the standard of care for the majority of aortic stenosis patients.

TAVR outcomes depend on providers from multiple disciplines, including cardiology, cardiac surgery, radiology, and anesthesiology. Of particular importance is the fundamental choice between general anesthesia (GA) and conscious sedation (CS) and its potential impact on outcomes [5-6].

Conscious sedation is defined as “a drug-induced depression of consciousness during which patients respond purposefully to verbal commands and are able to maintain a patent airway.” General anesthesia involves “drug-induced loss of consciousness during which patients are not arousable, even by painful stimulation. The ability to independently maintain ventilatory function is often impaired. Patients often require assistance in maintaining a patent airway, such as mechanical ventilation" [7].

In the early days of TAVR, GA was used for all cases, however, the excessive depth of anesthesia used in GA has been associated with higher mortality and delirium in several large studies [8-9]. It was quickly realized that CS for TAVRs was a feasible choice, with initial successful reports from Europe followed by several case series from the United States [10]. Recently, multiple small observational studies have examined the safety and efficacy of anesthetic choice and found that CS is associated with shorter procedural times and hospital stay. It also minimizes the use of inotropes without compromising procedural success [11-12]. Some studies, however, did not show any difference in short-term mortality between CS and GA [10].

Although most of the data have shown improved clinical outcomes with CS; consensus regarding the impact on cost between CS and GA for TAVR is still evolving [13]. Some studies have shown improved cost with the use of CS in TAVR [12-13], however, they lack a detailed breakdown on cost and the majority of the cost data were not adjusted for inflation.

In our cohort, we attempt to compare the clinical outcomes between CS and GA for TAVR while analyzing the detailed cost difference between the two groups.

## Materials and methods

Patient population and study design

We did a retrospective chart review of 434 patients who received TAVR at OSF Saint Francis Medical Center at Peoria, Illinois, between December 2012 and April 2018. Patients with an aborted procedure and those requiring a cardiopulmonary bypass or surgical conversion (16 patients) were excluded. The final sample size was 418. Patients with missing variables (18) were excluded for adjusted logistic regression of clinical variables, where the sample size was 400.

Institutional review board approval was obtained from the office of human research at the University of Illinois Chicago at Peoria, IL. Considering the retrospective nature of this study, a consent waiver was approved. All patients undergoing TAVR were deemed as intermediate or high-risk for SAVR by the local cardiothoracic surgery team based on the Society of Thoracic Surgeons (STS) score.

Clinical, electrocardiographic, and echocardiographic data were extracted retrospectively, and every patient had a baseline electrocardiogram (EKG) and echocardiogram done before TAVR. The clinical variables analyzed included: age, gender, body mass index (BMI), STS score, history of hypertension, diabetes, prior myocardial infarction, heart failure with different New York Heart Association (NYHA) functional classes , atrial fibrillation or flutter, smoking, chronic lung disease, use of home oxygen, and renal disease requiring dialysis. In addition, we looked at the pre-procedure hemoglobin and creatinine levels. Frailty was calculated through a composite score that included Kansas City Cardiomyopathy 12-question survey score (KCCQ12) and serum albumin level (<3.5 mg/dL).

Echocardiographic variables included the left ventricular internal diameter measured at systole and diastole (LVIDs/LVIDd) and ventricular septal wall thickness.

Outcome comparison

The primary outcomes included hours of stay in the intensive care unit (ICU) and length of stay (LOS) in days, readmission, and mortality at 30 days after TAVR.

Secondary outcomes comprised the cost differences between the two groups. We calculated the direct, indirect, and total costs of TAVR admission.

The direct cost was the cost incurred by individual patient care; it varied with patient volume. It was a combination of "direct fixed" and "direct variable cost." The direct fixed cost did not change with the patient's complexity and length of stay; it included fixed labor, e.g., salaries and wages; fixed benefits of staff, e.g. health insurance; Federal Insurance Contributions Act (FICA), 401k, and fixed purchase services, e.g., maintenance contracts, pharmaceuticals, equipment maintenance, and offset expenses. The direct variable cost was a combination of labor and benefits, implants, e.g., TAVR valves, pharmacy, blood supply, lab supplies, repair, and maintenance, and would be higher for complex patients and those with a longer stay.

Indirect costs covered the overhead cost allocated to each case; these costs are not volume sensitive and cannot be impacted at the bedside, e.g. facility costs, housekeeping, maintenance, and information technology. Total cost is a combination of both direct and indirect costs.

Statistical analysis

Patients were divided into two groups based on anesthesia status. Baseline characteristics and clinical data were compared among groups. Continuous data were represented as mean ± SD and categorical data as proportions. The T-test was used to compare continuous variables and Chi-square tests for categorical variables.

Adjusted statistical analyses were conducted for clinical and economic outcomes. For the readmission and mortality outcome, a logistic regression analysis was conducted. For ICU hours and length of stay, a generalized linear model with log link and Poisson distribution was used. For the cost outcome (direct cost, indirect cost, and total cost), the generalized linear model with log link and gamma distribution was used. All cost variables were inflated to the 2018 US dollar using the Inpatient Hospital Service Consumer Price Index (CPI).

The key covariate is an anesthesia type. Common covariates for adjusted analysis included age, male gender, smoking status, post-TAVR pacemaker implantation, body mass index (BMI), hemoglobin (pre-procedure), creatinine (pre-procedure), left ventricular internal diameter-systolic (LVIDs), left ventricular internal diameter-diastolic (LVIDd), septal wall thickness, valve type, valve size, access type, prior New York Heart Association (NYHA) class, chronic obstructive pulmonary disease (COPD), diabetes, dialysis, home O_2_, immunosuppression, prior myocardial infarction (MI), prior two-week heart failure, hypertension, atrial fibrillation or flutter, conduction defect, and year of procedure.

All calculations were performed using STATA 12 (STATA Corp, Texas) and a p-value of less than 0.05 will be considered statistically significant.

## Results

Baseline characteristics

Of the 418 patients in this study, CS was given to 194 (46.4%) patients while GA was given in 224 (53.6%) patients. The GA group had a comparatively older age (81.8 vs. 80.0; p=0.03) and a higher average STS score (8.4 vs 5.7; p<0.001). Gender did not differ across the two groups (males: 54% vs. 50.5%: p=0.475) (Table 1).

**Table 1:** Baseline characteristics Proportion represent the columns. STS: Society of Thoracic Surgery; LVIDs/LVIDd: left ventricular internal diameter measured at systole and diastole; NYHA: New York Heart Association; HF: heart failure

Variables	All Sample (N=418)	Conscious Sedation (N=194)	General Anesthesia (N=224)	
Continuous variables	Mean (SD)	Mean (SD)	Mean (SD)	P-value*
Age	80.9 (8.5)	80.0 (8.8)	81.8 (8.1)	0.033
STS score	7.1 (5.3)	5.7 (3.8)	8.4 (6.0)	<0.001
Hb (pre-procedure)	11.9 (1.7)	12.1 (1.7)	11.7 (1.7)	0.012
Creatinine (pre-procedure)	1.3 (0.9)	1.3 (0.8)	1.3 (0.9)	0.586
LVIDs	3.2 (0.8)	3.2 (0.8)	3.2 (0.8)	0.151
LVIDd	4.6 (0.7)	4.6 (0.7)	4.5 (0.7)	0.125
Categorical variables	# of sample (proportion)	# of sample (proportion)	# of sample (proportion)	P value*
Male	219 (52.4%)	98 (50.5%)	121 (54.0%)	0.475
Smoker	24 (5.7%)	10 (5.1%)	14 (6.3%)	0.631
Prior pacemaker	36 (8.6%)	15 (7.7%)	21 (9.4%)	0.550
Post pacemaker	19 (4.6%)	11 (5.7%)	8 (3.6%)	0.304
Valve type				<0.001
Sapien	49 (11.7%)	0 (0.0%)	49 (21.9%)	
Sapien XT	79 (18.9%)	1 (0.5%)	78 (34.8%)	
Sapien 3	290 (69.4%)	193 (99.5%)	97 (43.3%)	
Body Mass Index				0.347
Underweight (<25)	110 (26.3%)	50 (25.8%)	60 (26.8%)	
Normal (25~ <30)	143 (34.2%)	59 (30.4%)	84 (37.5%)	
Overweight (30~ <35)	90 (21.5%)	46 (23.7%)	44 (19.6%)	
Obesity (>=35)	75 (17.9%)	39 (20.1%)	36 (16.1%)	
Septal wall thickness (cm)				0.542
<1.1	83 (19.9%)	41 (21.1%)	42 (18.7%)	
>=1.1	335 (80.1%)	153 (78.9%)	182 (81.3%)	
Access type				<0.001
Femoral	364 (87.1%)	194 (87.1%)	170 (75.9%)	
Trans Aortic	24 (5.7%)	0 (0.0%)	24 (10.7%)	
Trans Apical	24 (5.7%)	0 (0.0%)	24 (10.7%)	
Trans Iliac	4 (0.9%)	0 (0.0%)	4 (1.8%)	
Subclavian	2 (0.5%)	0 (0.0%)	2 (0.9%)	
Prior-NYHA 2 category				0.102
I-II	60 (14.3%)	22 (11.3%)	38 (17.0%)	
III-IV	358 (85.7%)	172 (88.7%)	186 (83.0%)	
Chronic lung disease				0.125
None	229 (54.8%)	118 (60.8%)	111 (49.5%)	
Mild	89 (21.3%)	37 (19.1%)	52 (23.2%)	
Moderate	66 (15.8%)	27 (13.9%)	39 (17.4%)	
Severe	34 (8.1%)	12 (6.2%)	22 (9.8%)	
Diabetes	170 (40.7%)	82 (42.3%)	88 (39.3%)	0.536
Dialysis	14 (3.4%)	5 (2.6%)	9 (4.0%)	0.414
Home O2	30 (7.2%)	6 (3.1%)	24 (10.7%)	0.003
Immunosuppression	35 (8.4%)	13 (6.7%)	22 (9.8%)	0.251
Prior MI	124 (29.7%)	61 (31.4%)	63 (28.1%)	0.459
Pr 2wk HF	122 (29.2%)	22 (11.3%)	100 (44.6%)	<0.001
Hypertension	387 (92.6%)	176 (90.7%)	211 (94.2%)	0.176
A fib/flutter	159 (38.0%)	77 (39.7%)	82 (36.6%)	0.517
Conduction defect	223 (53.4%)	95 (48.9%)	128 (57.1%)	0.095

**Figure 1:**
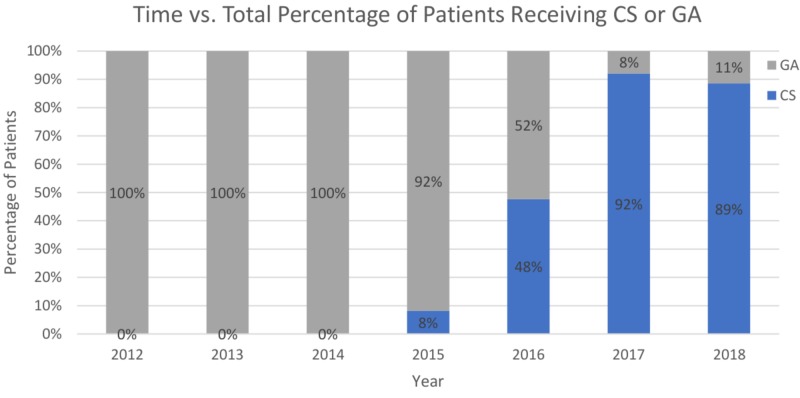
Chronological utilization trend between conscious sedation or general anesthesia in TAVR CS: conscious sedation; GA: general anesthesia, TAVR: transcatheter aortic valve replacement

It is noteworthy that over the years, our center used CS more often as compared to GA (Figure 1).

Clinical and procedural variables

The GA group saw more patients with symptomatic heart failure within two weeks prior to TAVR (44.6% vs. 11.3%; p<0.001) and those requiring supplemental home oxygen (10.7% vs. 3.1%; p=0.003). The average pre-procedural hemoglobin was slightly greater in the CS group (12.1 vs. 11.7; p=0.012). No other clinical variables were statistically significant.

Regression analysis

**Figure 2:**
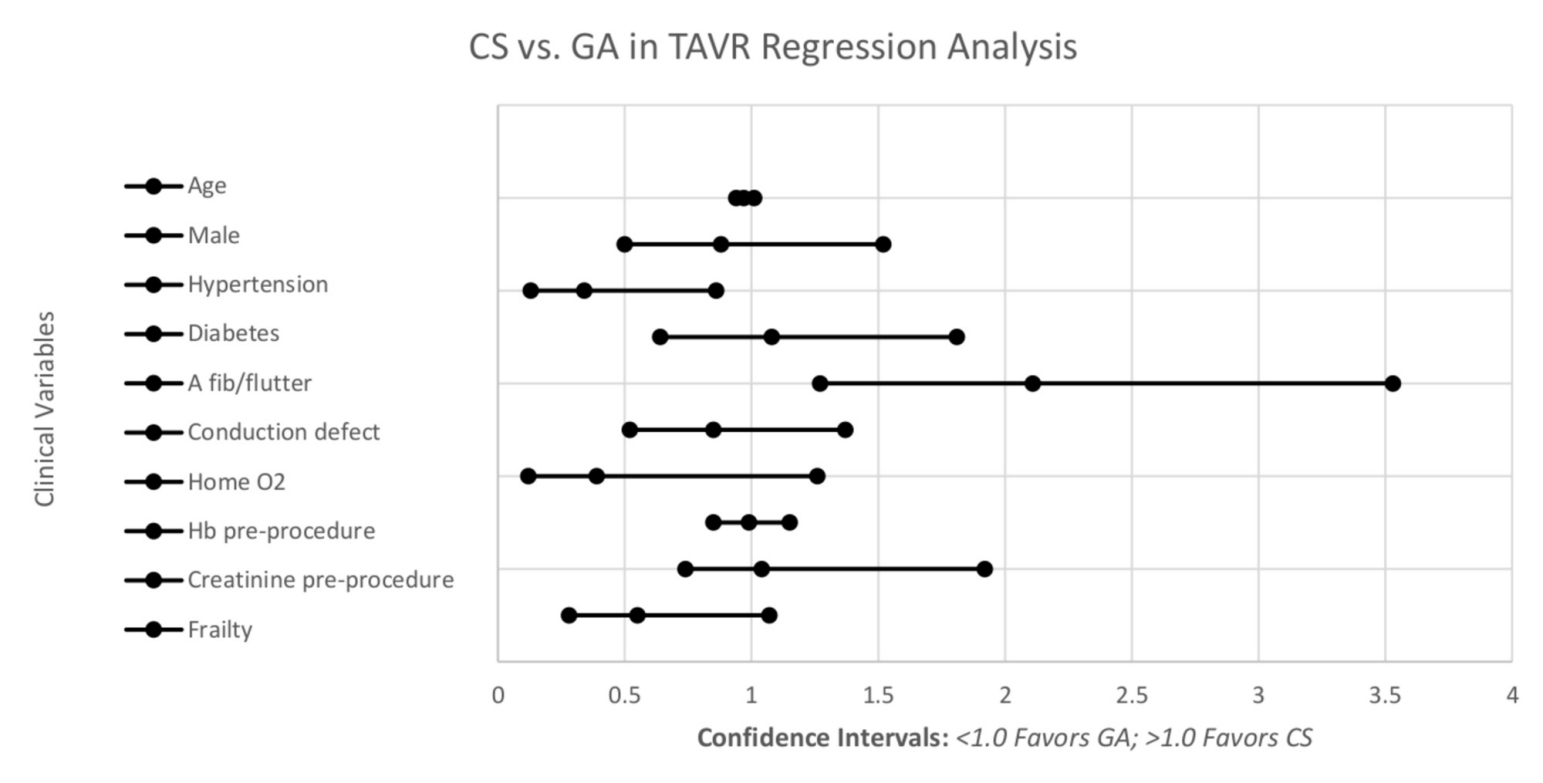
Logistic regression adjusted for clinical variables CS: conscious sedation; GA: general anesthesia; TAVR: transcatheter aortic valve replacement

Looking at logistic regression adjusted for other clinical variables (Figure 2, Table 2), the odds of being in the CS group was 2.28 times more likely for patients with a prior history of NYHA class III and IV (OR: 2.28; CI: 1.17 - 4.46; p=0.016). Patients with hypertension were less likely to receive CS (OR: 0.34; CI: 0.13 - 0.86; p=0.022). Patients with atrial fibrillation/flutter had increased odds of receiving CS (OR: 2.11; CI: 1.27 - 3.53; p=0.004). Symptomatic heart failure within two weeks prior to TAVR indicated greater odds of receiving GA (OR: 0.13; CI: 0.07 - 0.26; p<0.001). Frail patients had higher odds of receiving GA (OR: 0.55, CI: 0.07-0.28, p=1.07).

**Table 2:** Adjusted logistic regression result (outcome: general anesthesia/conscious sedation, n=400) The following variables omitted due to collinearity: valve size, valve type, dialysis, and year. Sample size reduced to 400 due to missing values of frailty in 18 observations.

Covariates	Odds Ratio	P value	95% Confidence Interval	
Frailty	0.55	0.077	0.28	1.07
Age	0.97	0.149	0.94	1.01
Male	0.88	0.636	0.50	1.52
Smoker	1.02	0.972	0.35	2.99
STS score	0.95	0.154	0.89	1.02
Prior pacemaker	1.79	0.192	0.75	4.29
BMI (Ref: Normal)				
Underweight	1.68	0.100	0.91	3.13
Overweight	1.39	0.323	0.72	2.68
Obese	1.58	0.236	0.74	3.38
Hb pre-procedure	0.99	0.902	0.85	1.15
Creatinine (pre-procedure)	1.04	0.773	0.78	1.41
LVIDs	1.19	0.480	0.74	1.92
LVIDd	1.00	0.992	0.58	1.71
Septal wall thickness >=1.1 (Ref: <1.1)	0.76	0.378	0.41	1.41
Prior NYHA III-IV	2.28	0.016	1.17	4.46
Chronic lung disease (Ref: None)				
Mild	0.49	0.027	0.26	0.92
Moderate	0.62	0.185	0.30	1.26
Severe	0.65	0.404	0.24	1.77
Diabetes	1.08	0.783	0.64	1.81
Home O2	0.39	0.116	0.12	1.26
Immunosuppression	0.64	0.342	0.26	1.60
Prior MI	1.32	0.319	0.77	2.26
Prior 2-week HF	0.13	<0.001	0.07	0.26
Hypertension	0.34	0.022	0.13	0.86
A fib/flutter	2.11	0.004	1.27	3.53
Conduction defect	0.85	0.498	0.52	1.37
Constant	17.23	0.198	0.23	1309.28

Multivariable analysis

Patients who received CS during TAVR stayed in the ICU about 10 hours less than GA patients (31.5 vs. 41.6 hours, p<0.001). CS patients were discharged almost a day earlier than GA patients (2.9 vs. 3.8 days, p=0.010) (Table 3).

**Table 3:** Multivariate analysis All costs were converted to 2018 US Dollar using the Inpatient Hospital Service Consumer Price Index (CPI). Covariates included gen/conscious sedation, post pacemaker, age, male, smoking status, Society of Thoracic Surgery (STS) score, body mass index (BMI), Hb pre-procedure, creatinine (pre-procedure), left ventricular internal diameter (systolic) (LVIDs), left ventricular internal diameter (diastolic) (LVIDd), septal wall thickness, valve type, valve size, access type, prior New York Heart Association (NYHA), chronic lung disease, diabetes, dialysis, home O_2_, immunosuppression, prior myocardial infarction (MI), prior two-week heart failure, hypertension, atrial fibrillation/flutter, conduction defect, and year. 1 Generalized linear model (GLM) with log link and Poisson distribution adjusting for the above covariates. 2 Generalized linear model (GLM) with log link and gamma distribution adjusting for the above covariates. 3 Two-part model with logit and GLM due to lots of zero values adjusted for the above covariates.

Outcomes	Conscious Sedation (N=194) Mean (95% CI)	General Anesthesia (N=224) Mean (95% CI)	Difference Mean (95% CI)	P-value
ICU hours^1^	31.5 (30.3, 32.7)	41.6 (40.5, 42.8)	-10.1 (-12.2, -8.1)	<0.001
Length of stay (days)^1^	2.9 (2.5, 3.3)	3.8 (3.4, 4.1)	-0.8 (-1.5, -0.2)	0.010
Total cost^2^	$72,809 (69252, 76366)	$71,497 (68424, 74570)	$1,312 (-4460, 7085)	0.656
Direct cost^2^	$47,703 (45484, 49921)	$46,815 (44931, 48698)	$888 (-2683, 4459)	0.626
Pharmacy	$619 (363, 874)	$939 (726, 1151)	-$320 (-661, 21)	0.066
Blood^3^	$426 (119, 733)	$363 (258, 469)	$62 (-280, 405)	0.720
Laboratory	$484 (410, 558)	$708 (643, 773)	-$223 (-335, -112)	<0.001
Room	$3,553 (2891, 4215)	$4,517 (3930, 5103)	-$963 (-2002, 75)	0.069
Supply^3^	$147 (61, 233)	$189 (149, 229)	-$42 (-131, 46)	0.350
Therapy	$536 (409, 663)	$914 (795, 1034)	-$378 (-570, -186)	<0.001
Imaging	$411 (334, 488)	$457 (387, 528)	-$46 (-171, 79)	0.471
Miscellaneous Cost	$40,528 (39401, 41654)	$38,704 (37693, 39716)	$1,823 (-39, 3686)	0.055
Indirect cost^2^	$25,106 (23719, 26493)	$24,676 (23436, 25916)	$429 (-1855, 2715)	0.712

Cost analysis

There was no statistical difference in cost between the two groups. The average total cost for the CS group was actually slightly higher than the GA group with a difference of $1,312 per patient ($72,809 vs. $71,497: p=0.656). Direct and indirect costs were also higher for the CS group (Table 3).

However, taking a look at the breakdown, the only subcategories of the cost that were high for CS patients were blood products (by $62; p=0.72) and “miscellaneous costs” (by $1,823; p=0.055) (Table 4). The miscellaneous cost included cost not related to the TAVR procedure directly but was incurred because of the patient's other co-morbidities, mostly non-cardiac. All other subcategories of cost were actually higher for the GA group with “laboratory” and “therapy” costs being significantly high (p<0.001).

**Table 4:** Miscellaneous cost categories IV: intravenous; PICC: peripherally inserted central catheter; RN: registered nurse

Miscellaneous Cost
Surgery - General (Major)
Gastro-Intestinal Services
Pulmonary Intervention Lab
Psychiatric Emergency Care
Recovery Room services
IV Nutritional Support
Emergency services
ER Physicians
Electroencephalography
Hematology services
Urology Clinic
Pulmonary Function Services
PICC RN

Readmission and mortality

Although the CS group had a better readmission rate at 30 days (OR: 0.56; CI: 0.25 - 1.27; p=0.168) and 30 day mortality after procedure (OR: 0.63; CI: 0.98 - 4.01: p=0.623), these figures were not statistically significant (Table 5).

**Table 5:** Adjusted logistic regression result (n=418) Covariates included general anesthesia/conscious sedation, age, male, smoking status, STS score, body mass index (BMI), Hb pre-procedure, creatinine (pre-procedure), left ventricular internal diameter (systolic) (LVIDs), left ventricular internal diameter (diastolic) (LVIDd), septal wall thickness, valve type, valve size, prior New York Heart Association (NYHA), chronic lung disease, diabetes, dialysis, home O_2_, immunosuppression, prior myocardial infarction (MI), prior two-week heart failure, hypertension, atrial fibrillation/flutter, and conduction defect. The following variables were excluded due to multicollinearity: access type and year.

Outcomes	Variable	Odds Ratio	P-value	95% Confidence Interval	
30-day readmission	Conscious Sedation	0.56	0.168	0.25	1.27
30-day mortality	Conscious Sedation	0.63	0.623	0.98	4.01

## Discussion

Our data reflects that the use of conscious sedation in TAVR is associated with significant improvement in the clinical outcome of ICU stay and the total length of stay in the hospital. The CS group also fared better for readmission and mortality within 30 days of TAVR, however, the difference was not statistically significant. These trends for clinical outcomes are consistent with prior literature [10-11] and validate our findings. Frohlich and colleagues reported comparable clinical outcomes for TAVR with the use of CS when compared with GA [10].

There are limited data available on the cost comparison between CS and GA for TAVR patients. Some studies reflect a better cost for TAVR when using CS. Our cohort showed no statistical difference in cost for CS vs. GA in TAVR. Total, direct, and indirect cost for the CS group was actually slightly higher than the GA group. These findings are interesting, especially considering the fact that ICU hours and LOS were significantly lower for the CS group.

Therefore, we acquired a breakdown of cost in different subcategories from the department of finance at our institution (Table 3). This explains that although the total, direct, and indirect costs of TAVR are slightly higher for the CS group, the majority of the subcategories are cost-effective for patients in the CS group; these include pharmacy, laboratory, room, supply, physical therapy, and imaging. The major difference is created by "miscellaneous cost," which is higher for the CS group by $1823 per patient. The breakdown of this category in Table 4 shows that the majority of these expenses are not related to the actual TAVR procedure and are influenced by other needs of the patient during the admission, which is usually patient-specific, based on their co-morbidities. For example, these patients required hematology, pulmonary, psychiatry, neurology, and urology services. It is noteworthy that after excluding the miscellaneous category, the difference in cost may become beneficial for the CS group, however, it will not achieve statistical significance.

A significantly lower cost of laboratory use by CS patients (Table 3) reflects that the CS group, which has a shorter LOS did not use laboratory testing as much; possibly reflecting the use of daily laboratory testing in the GA group. Physical therapy services were utilized less frequently by the CS group, which coincides with their shorter LOS. In addition, these patients were generally less frail (Figure 1) as compared with the GA group.

It is noteworthy, that age, STS score, and pre-procedure hemoglobin that were significantly different between the two groups initially (Table 1), when adjusted for other clinical variables (Table 3) did not show any statistical significance.

Strengths and limitations

Our institution allowed the publication of actual cost data. Such a detailed analysis of cost was not found in the literature published so far. This data is of significant validity as the cost was adjusted for inflation to compare different years of service, which is not seen in some prior studies [13].

This was a single-center, retrospective review of prospectively maintained databases and, as such, has certain inherent limitations. Given the novelty of TAVR as a procedure, our study is limited to a five-year span and relatively small sample size. As there is a transition from first-generation to third-generation TAVR valves over the study period, we did not have the cost difference available for the particular prosthesis used for TAVR.

## Conclusions

When compared with general anesthesia, conscious sedation improves morbidity in the form of ICU stay and the total length of hospital stay for TAVR patients in our cohort. These findings are similar to the prior available evidence.

Although the cost for the majority of subcategories is lower for patients who received conscious sedation, however, we did not find a significant difference in cost for TAVR admission when comparing conscious sedation with general anesthesia. The feasibility of conscious sedation coupled with better clinical outcomes has made it a standard of care for the minimally invasive TAVR procedure.
